# GATA5 mutation homozygosity linked to a double outlet right ventricle phenotype in a Lebanese patient

**DOI:** 10.1002/mgg3.190

**Published:** 2015-12-20

**Authors:** Kameel Kassab, Hadla Hariri, Lara Gharibeh, Akl C. Fahed, Manal Zein, Inaam El‐Rassy, Mona Nemer, Issam El‐Rassi, Fadi Bitar, Georges Nemer

**Affiliations:** ^1^ Department of Biochemistry and Molecular GeneticsAmerican University of BeirutBeirutLebanon; ^2^ Department of BiochemistryUniversity of OttawaOttawaOntarioCanada; ^3^ Department of GeneticsHarvard Medical School and Department of Internal MedicineMassachusetts General HospitalBostonMassachusetts; ^4^ Department of SurgeryAmerican University of BeirutBeirutLebanon; ^5^ Department of Pediatrics and Adolescent MedicineAmerican University of BeirutBeirutLebanon

**Keywords:** Congenital, GATA5, heart, homozygous, recessive, transcription

## Abstract

**Background:**

GATA transcription factors are evolutionary conserved zinc finger proteins with multiple roles in cell differentiation/proliferation and organogenesis. GATA5 is only transiently expressed in the embryonic heart, and the inactivation of both *Gata5* alleles results in a partially penetrant bicuspid aortic valve (BAV) phenotype in mice. We hypothesized that only biallelic mutations in *GATA5* could be disease causing.

**Methods:**

A total of 185 patients with different forms of congenital heart disease (CHD) were screened along 150 healthy individuals for *GATA4, 5*, and *6*. All patients' phenotypes were diagnosed with echocardiography.

**Results:**

Sequencing results revealed eight missense variants (three of which are novel) in cases with various conotruncal and septal defects. Out of these, two were inherited in recessive forms: the p.T67P variant, which was found both in patients and in healthy individuals, and the previously described p.Y142H variant which was only found in a patient with a double outlet right ventricle (DORV). We characterized the p.Y142H variant and showed that it significantly reduced the transcriptional activity of the protein over cardiac promoters by 30–40%.

**Conclusion:**

Our results do prove that p.Y142H is associated with DORV and suggests including *GATA5* as a potential gene to be screened in patients with this phenotype.

## Introduction

Heart development is a highly evolutionary conserved event during embryogenesis attributed largely to the dependence of other organs on its proper function for their own growth and maturation (Olson and Srivastava 1996; Srivastava and Olson 2000; McFadden and Olson 2002; Calderon Colmenero 2007; Nemer 2008). The proper spatio‐temporal expression of key proteins and microRNAs during a very specific window of time represents thus an obligatory roadmap for the formation of a functional heart. Any deviation from this path would result in major defects that can either lead to congenital heart diseases (CHD) or spontaneous termination of pregnancy. Congenital heart diseases in fact represent the most common congenital defects accounting for up to 25% of total neonatal structural and functional defects occurring in approximately 1% of all newborns (Lage et al. 2012). Despite tremendous knowledge gained from human genetic studies and animal models in the last two decades, we still do not have a reliable map of all the genetic and epigenetic events that cause CHDs. More recently, exome sequencing of trios' de novo mutations and copy number variations (CNVs) have been implicated in sporadic CHD (Yuan et al. 2013; Glessner et al. 2014) (Fahed et al. 2013).

Numerous transcriptional regulators, structural proteins, and signaling molecules have been implicated in the process of cardiogenesis (Nemer 2008). Defects in those major regulator proteins have been highly linked to many CHDs. The GATA family of transcription factors are evolutionary conserved zinc finger DNA‐binding proteins (Temsah and Nemer 2005). Three of the mammalian GATA proteins, GATA4, 5, and 6 are highly expressed in both the cardiac mesoderm and underlying endoderm during embryonic development. Their nonredundant role in different aspects of cardiac morphogenesis was highlighted in the differential phenotypes obtained from the inactivation of their encoding genes in mice (Molkentin et al. 1997; Morrisey et al. 1998; Laforest et al. 2011).

Despite being expressed only in the mammalian embryonic heart, unlike *GATA4* and *GATA6*,* GATA5* was not put onto the CHD map until the publication by Laforest et al. (2011) of the results of the inactivation of the gene in mice. In vivo, the mammalian GATA5 expression is restricted spatio‐temporally to the endocardial cells and endocardial cushions of the outflow tract (OFT) and atrio‐ventricular canal (AVC), and gets downregulated shortly after the formation of the valves (Nemer and Nemer 2003). In vitro, the knock‐down of *Gata5* expression in TC13 cells leads to inhibition of endocardial differentiation and point out to an essential role for the combinatorial interaction of GATA5 with the nuclear factor of activated T‐cells (NFATC1) in regulating endocardial specific gene expression (Nemer and Nemer 2002). Inactivation of both copies of the gene in mice leads to bicuspid aortic valve (BAV) with partial penetrance confirming thus, the specific nonredundant role of *Gata5* in heart development. Interestingly, the same phenotype was obtained using the Tie2‐Cre conditional knock‐out confirming an autonomous role in endocardial and endocardial cushion cells (Laforest et al. 2011). In addition, mice that are double heterozygous for both *Gata5* and *Gata4*, or for both *Gata5* and *Gata6* died embryonically or prenatally, mostly due to severe defects of the OFT including double outlet right ventricle (DORV) and ventricular septal defect (VSD) (Laforest and Nemer 2011). Defects in the OFT represent 20–30% of all CHDs and include in addition to DORV, persistent truncus arteriosus (PTA), transposition of the great arteries (TGA), tetralogy of fallot (TOF), and aortic and pulmonary valve stenosis (Benson et al. 1996; Restivo et al. 2006). The relative complex phenotypes associated with the OFT defects reflect the numerous molecular pathways involved in its formation and the interaction of both cardiac and neural crest cells (Waldo et al. 2001; Hutson and Kirby 2007; Rochais et al. 2009; Kelly 2012; Zaffran and Kelly 2012). The conserved role of GATA5 in heart development and in particular the phenotypes observed in mice with total or partial loss of functions prompted researchers to investigate its role in CHDs. So far, 12 articles were published showing different *GATA5* sequence variants to be correlated with different forms of CHDs ranging from atrial fibrillation (AF), to TOF, VSD, and BAV (Gu et al. 2012; Jiang et al. 2012, 2013; Padang et al. 2012; Wei et al. 2012, 2013a,b; Yang et al. 2012; Wang et al. 2013; Bonachea et al. 2014; Shi et al. 2014; Zhang et al. 2014). In all the published findings, the variants are however heterozygous, and in most of the cases genotyping of the parents was not performed making it difficult to establish a causality link.

Studying such variations in genetically homogeneous populations, like the Lebanese, offers better opportunities to correlate phenotypes to genotypes. We have previously shown that close to 20% of the parents of children affected with CHD in our registry at the American University of Beirut Medical Center (AUBMC) are first cousins (Bitar et al. 1999, 2001). We therefore hypothesize that in most of these cases recessive mutations would explain the underlying phenotypes. Since the mouse *Gata5* model of BAV entails losing both alleles, we predicted that *GATA5* variants on both alleles would be associated with BAV or other forms of CHDs in our patients. We have therefore screened 185 patients with different forms of CHDs, and identified two variants affecting both alleles of the patients, and three novel variants affecting only one allele. One of the recessive variants, p.T67P, is however found also in healthy individuals and thus was considered a natural variant, whereas the other, p.Y142H (rs111554140) was previously described as being responsible for BAV and was not encountered in any of the 150 healthy controls included in this study. Our in vitro analysis of the mutated protein showed that the p.Y142H is responsible for the DORV phenotype observed in the affected patient but is not associated with BAV since both parents carrying one copy of the altered allele are healthy. In addition, the three novel variants found in the patients are probably not directly linked to the observed phenotype since they are inherited from one healthy parent, but might contribute to the observed phenotype in combination with other variants in cardiac enriched genes functionally interacting with *GATA5*.

## Materials and Methods

### Patient recruitment and clinical examinations

The study was approved by the institutional review board at the American University of Beirut (protocol number: Bioch.GN.01/05). All patients, their legal guardians, and family members signed an informed consent form before being enrolled in the study. Patients presenting to the Department of Pediatrics and Adolescent Medicine, Division of Cardiology, at the American University of Beirut Medical Center with a diagnosis of CHD were serially recruited in the study. A total of 185 patients with various forms of CHDs were enrolled. A total of 150 healthy unrelated individuals were also recruited as controls by clinical history. Standard clinical evaluation included a complete physical exam, electrocardiography (ECG), and two‐dimensional (2D) transthoracic echocardiography (TTE) with color Doppler were obtained. Family consanguinity history was utilized in constructing pedigrees after interviewing all patients and their parents. Patients with documented syndromes and gross chromosomal abnormalities were excluded from the study.

### Genetic analysis

A sample of peripheral venous blood was collected from each participant and genomic DNA was extracted from the white blood cells using the Qiagen Blood‐Midi kit (Qiagen Science Inc., Germantown, MD), following the manufacturer's protocol. Primers to amplify all coding exons of the human *GATA4*,* GATA5*, and *GATA6* genes were designed using the Primer3 software (http://frodo.wi.mit.edu/cgi-bin/primer3/primer3_www.cgi) in the intronic regions (Table S1). Amplification by polymerase chain reaction (PCR) was done using the Phusion polymerase high‐fidelity master mix (F‐548S) on a Pico machine (Finnzymes, Espo, Finland). The amplicons were resolved on a 1.5% agarose gel. Purification from gel was performed using the Gel Extraction kit following the manufacturer's protocol (peqGOLD Gel Extraction Kit, PeqLab, Erlangen, Germany). The purified bands were quantified using a NanonDrop (Thermo Fisher Scientific Inc., Waltham, MA) and examined by gel electrophoresis to ensure quality.

### DNA sequencing

DNA sequencing was done using the four‐reactions/one gel system of the chain‐terminator method. Sequencing was done on an ABI 3100A machine, and analysis of the results was carried out using the data collection software v1.5 from Applied Biosystems Inc. (Foster City, CA). Sequencing was done twice to confirm the mutations using the forward and reverse primers.

### Plasmids

An expression vector harboring the human *GATA5* cDNA fused to a Myc‐tagged epitope was purchased from OriGene (catalog number RC218399). Amplification of the coding region was carried out by PCR, and the resulting amplicon subcloned into the pCGN‐HA‐tagged plasmid using the restriction enzymes XbaI and BamHI. Site‐directed mutagenesis was carried out introducing the Y142H mutation into the wild‐type *GATA5* using Site‐Directed Mutagenesis kit from FINNZYMES (product code: F‐541). The resulting amplicon was ligated and transformed into XL‐1 Blue competent bacteria. The generated plasmid was extracted and sequenced thereafter to confirm the mutation incorporation and exclude any other miscellaneous mutation entry.

### Cell lines

HEK 293T cells (human embryonic kidney cells) and HeLa cells (human cervical cancer cells) were cultured and maintained in Dulbecco's modified Eagle medium (DMEM) supplemented with 10% fetal bovine serum (FBS), 1% penicillin/streptomycin, and 1% sodium pyruvate. Incubation was carried out in a humid atmosphere 5% CO_2_ at 37°C as previously described.

### Immunofluorescence

HeLa cells were plated in 12‐well Costar culture plates on cover slips with 100,000 cells/well. Transfections were done on the second day of plating using polyethylenimine (He et al. 2007). Briefly, 2 *μ*g of DNA per well were diluted in 150 *μ*L of culture medium, DMEM, in an eppendorf tube, vortexed and then 12 *μ*L of PEI were added on the mixture of DNA/DMEM, vortexed, for 3 sec and then incubated for 20 min at room temperature (RT). The prepared mixture was gently applied on the cells and the medium was replaced after three hours. Immunofluorescence was performed on transfected HeLa cells. The cells were first washed for 3 times with PBS 1X (phosphate‐buffered saline), and later fixed with 4% paraformaldehyde for 20 min. Fixed cells were blocked with 3% BSA/PBT (bovine serum albumin/phosphate buffer saline Tween) for 1 h. The primary antibody rabbit anti‐HA (Santa Cruz, Dallas, USA) was used for assessment of subcellular localization of GATA5 wild‐type and the mutant form. The primary antibodies were diluted in BSA/PBT and added to the cells with an overnight incubation at 4°C. The cells were then washed in PBT 3 times. Biotinylated donkey anti‐rabbit (Santa Cruz) was diluted 1:500 in BSA/PBT and added to the cells for 1 h at RT with shaking. After washing 3 times with PBT, cells were incubated with Streptavidin Texas red at RT. Staining for the nuclei were done using the Hoechst dye. The cells were mounted on a rectangular slide containing an antifading agent (DABCO), and the slides were examined using an Olympus BH‐2 microscope (St. Louis, USA). The nuclear versus cytoplasmic staining was conducted by three independent experiments with total assessment of 10‐fields/per experiment and a total of 125 cells for each mutant and wild‐type protein.

### Reporter gene assay

HeLa cells were transfected with the 1.6 kb human VEGF promoter or the −700 bp rat NPPA promoter or the mouse 1.6 kbp Nos3 or the minimal NPPB promoter with a GATA element coupled to the luciferase reporter together with the GATA5‐expressing vectors (encoding the wild‐type or mutated protein) as previously described. After 24 h, cells were washed with PBS (1X) and then lysed with 1X lysis buffer and left on the shaker for 20 min at RT. Luciferin (Promega, WI, USA, Cat # E 1501) was prepared according to the manufacturer's protocol. The lysed cells were transferred to a 96 well plate (Costar, St. Louis, USA) to which Luciferin was added and the signal was read immediately using the Ascent Fluoroscan in the Molecular Biology Core Facility at AUB.

### Protein overexpression and western blotting

Protein overexpression was achieved by transfecting human embryonic kidney cells (HEK293) with the corresponding GATA5 plasmid utilizing polyetheleneamine (He et al. 2007). Briefly, HEK293 cells were first plated in 100 mm culture plates (Corning) with 80% confluency determined by running a simultaneous green fluorescent protein (GFP) transfection assay. On the second day, 20 *μ*g DNA was added to an eppendorf tube; DMEM culture medium was added until 1 mL total volume. A quantity of 35 *μ*L PEI reagent was added to the eppendorf tube, and vortexed for 10 sec, followed by 20 min of incubation at RT. The mixture was applied over the cells and the cultures medium was changed after 3 h.

Nuclear protein extracts from HEK293 cells were obtained as previously described. A 30 *μ*L aliquots were stored at −80°C. For western blots, equal amounts of nuclear cell extracts (10 *μ*g protein) were resuspended in 5X Laemmli buffers. The samples were boiled for 3 min and run on a denaturing SDS‐PAGE for 1.5 h then transferred to a PVDF membrane (Amersham, UK). The membrane was blocked for 45 min in 2% nonfat dry milk. After blocking, the membrane was incubated with the primary antibody, Anti‐HA (against GATA5), and Anti‐Flag (against NFATC1). The antibody was diluted 1:1000 in 1% nonfat dry milk and the incubation was carried out overnight at 4°C. The membrane was later incubated with the secondary antibody conjugated with horseradish‐peroxidase, anti‐mouse, or anti‐rabbit‐ HRP, diluted by 1:40,000 ratios. Development was done using the Western Lightening Chemiluminescence Kit (Cat # NEL 103; Perkin Elmer, Akron, OH, USA). The protein bands were visualized by autoradiography and quantified using either the Image J or Image Lab (BioRad, CA, USA) software.

### Protein coimmunoprecipitation

Biochemical interaction between the GATA5 (wt and Y142H mutant) and GATA4/NFATC1/Tbx5 proteins was assessed utilizing protein coimmunoprecipitation assay. Briefly, 5 *μ*g of anti‐rabbit HA (Santa Cruz) antibodies were incubated with Dynabeads Protein G (Life Technologies, CA, USA) for 1 h at 4°C with PBS (1X + 0.001% tween 20). A mixture of proteins (representing GATA5 and NFAC1) with a total amount of 5X of that used in western blotting were added over the antibodies Dynbeads complex and incubated for two hours at RT. The resulting complex captured on the magnet was washed three times with PBS 1X. This was followed by a western blot as per regular protocol utilizing anti‐mouse Flag antibodies (Santa Cruz). After autoradiography and quantification by Image J, the PVDF membrane was stripped and probed with an anti‐HA rabbit antibody (Santa Cruz).

### Statistical analysis

The significance of luciferase transfection assay was assessed utilizing one‐way analysis of variance testing with significance defined as *P* < 0.05.

### Compliance

All methods were carried out in accordance with the approved guidelines.

## Results

One hundred ninety‐three patients with various forms of CHDs were recruited. Phenotypic classification was done according to the major phenotype assessed by echocardiography (Table 1). All parents were recruited as part of the study along with their affected child. One hundred and fifty healthy individuals were utilized as controls.

**Table 1:** Number of individuals recruited in the study with their primary phenotypes

Phenotype	Number (%)
ASD	26 (13.5)
AVC	7 (3.6)
CoA	13 (6.7)
PDA	8 (4.1)
PS	13 (6.7)
SV	14 (7.3)
TGA	11 (5.7)
PTA	3 (1.6)
TOF	30 (15.6)
VSD	19 (9.9)
AS/BAV	18 (9.3)
TA	4 (2.0)
PA	8 (4.1)
Other	11 (5.7)
Total	185 (100)

ASD, atrial septal defect; AVC, atrio‐ventricular canal; CoA, coarctation of the aorta; PDA, persistent ductus arteriosus; PS, pulmonary stenosis; SV, single ventricle; TGA, transposition of the great arteries; TOF, tetralogy of fallot; VSD, ventricular septal defect; AS/BAV, aortic stenosis/bicuspid aortic valve; TA, tricuspid atresia; PA, pulmonary atresia; PTA, persistent truncus arteriosus.

### Eight GATA5 variants in the Lebanese patients with CHD

**Figure 1:**
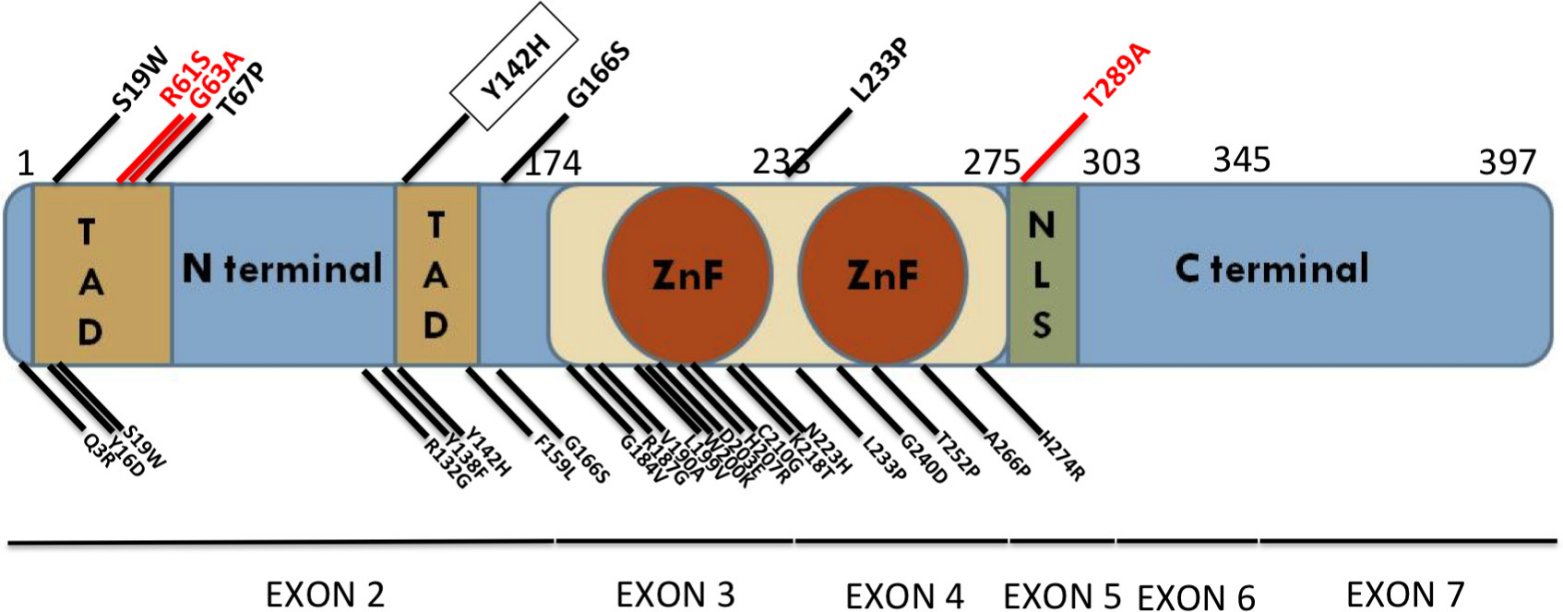
Schematic representation of the GATA5 protein with the different mutations. The diagram shows the different GATA5 domains with the amino acids boundaries representing the corresponding coding exons. The published missense mutations are shown below the diagram whereas the variants coming up from this study are shown above the diagram (in red, novel variants, and in black previously reported variants). ZnF, Zinc finger domain; TAD, transactivation domain; NLS, nuclear localization signal.

The coding exons of *GATA5* were screened in all of the 185 patients and the corresponding controls. We are reporting eight missense variants (Table 2 and Fig. 1) among which three are novel: p. R61S, p.G63A, and p.T289A. These variants occur only in one patient: p.R61S in a patient with VSD, p.G63A in a patient with pulmonary stenosis (PS), p.S19W in two patients with atrial septal defect (ASD) and TOF, and p.T289A in a patient with AVC malformation. Subsequent sequencing of the variants in the healthy parents of the patients showed that the variant is inherited in an autosomal dominant manner (Table 2). The same pattern of inheritance was also observed in the p.L233P variant in one patient with coarctation of the aorta (Bonachea et al. 2014) p.T67P variant, previously documented only in its heterozygous form (rs6142775) was found to be homozygous in a patient with valvular aortic stenosis, in a patient with atrio‐ventricular (AV) canal and L‐TGA, and in a patient with AVC. In addition, it was found in a heterozygous form in patients with CoA, VSD and persistent ductus arteriosus (PDA) (Table 2). This variant was also found in 4 healthy individuals out of 150 screened. The high minor allele frequency (MAF) of this variant in the Exome Aggregation Consortium (ExAC) database does not support a role for it in any forms of CHD, even in the homozygous form (Table 2). Finally, the previously reported BAV‐associated variant p.Y142H (rs111554140) (Padang et al. 2012), was found in one patient with a DORV phenotype, a small VSD, and a mild PS. Both parents were heterozygous for the p.Y142H variant, and echocardiograms show normal heart structure and function. This variant which was reported to have a frequency of 0.002 (Table 2) was not found in any of the 150 healthy Lebanese individuals recruited in this study, and is the first documented *GATA5* minor allele to be expressed in a homozygous manner suggesting a causal relationship with the DORV‐associated phenotype.

**Table 2:** List of DNA variants and their frequencies in the studied samples and general population

Locus	Nucleotide change	Amino acid change	dbSNP	Phenotype	No. patients (out of 193)	No. controls (out of 150)	No. parents (out of 386)	ExAC MAF	Polyphen 2 score (0–1)
20:61040938	T>C	T289A	–	AVC	1	0	1	0	1 (possibly damaging)
20:61048460	A>G	L233P	rs116164480	CoA	1	0	1	0.002020	0.723 (possibly damaging)
20:61050082	C>T	G166S	rs141950357	SV/TOF	2	0	2	0.01448	0.386 (benign)
20:61050154	A>G	Y142H	rs111554140	DORV	1	0	2	0.002035	1 (possibly damaging)
20:61050379	T>G	T67P	rs6142775	AS, CoA, VSD, PDA, AVC	6	4	9	0.2564	0 (benign)
20:61050383	G>C	G63A	–	PS	1	0	1	0	0.013 (benign)
20:61050385	C>A	R61S	–	VSD	1	0	1	0	0.013 (benign)
20:61050522	G>C	S19W	rs200383755	ASD/TOF	2	0	2	0.02969	1 (possibly damaging)

The locus indicates the chromosomal position. ExAc MAF, exome aggregation consortium minor allele frequency.

### The GATA5 p.Y142H variant does not affect the nuclear localization of the protein

**Figure 2:**
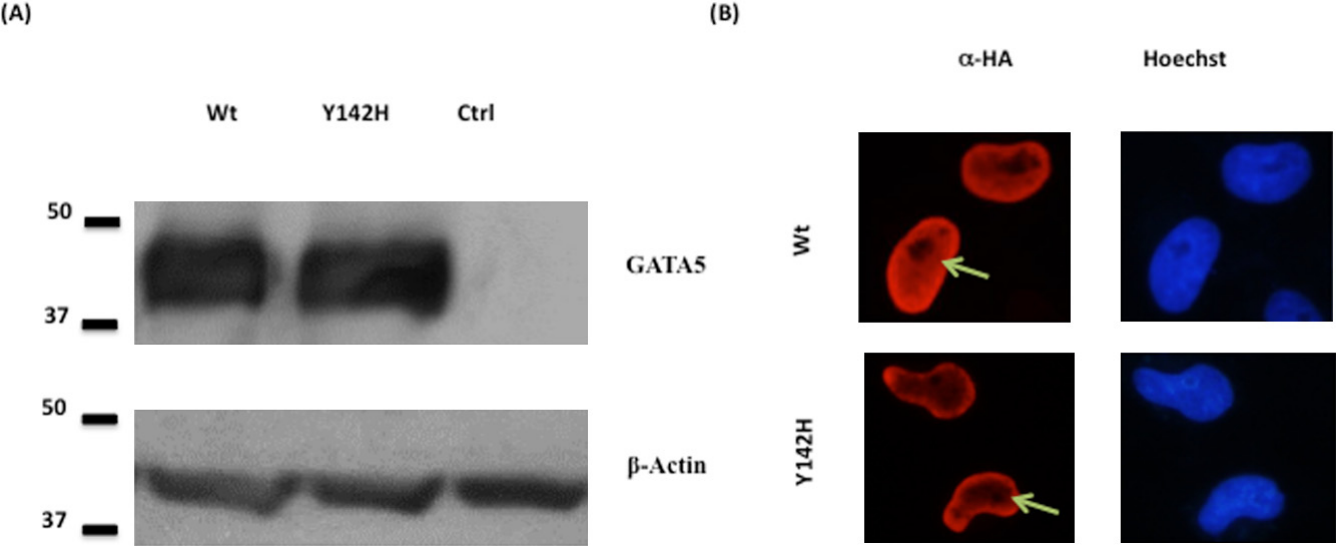
Effect of the p.Y142H mutation on the cellular localization of GATA5 protein. (A) GATA5 extracts from HEK 293 cells transfected with Wt GATA5 and mutated GATA5 (Y142H) were resolved on an SDS‐PAGE. Western blots showed equal amounts of expressed proteins as depicted by the anti‐HA antibody. *α*‐actin was used as a loading control. (Ctrl refers to nuclear extracts from mock‐transfected cells). (B) Immunofluorescence of HeLa cells transfected with plasmids encoding for the Wt GATA5 and GATA5 mutant (Y142H). The localization of GATA5 was visualized using an anti‐ HA antibody followed by a fluorescent secondary antibody. Nuclei of cells were visualized using the Hoechst dye (blue color). Wt and GATA5 mutant showed nuclear localization (red color). (Magnification ×60). Green arrow indicates nuclear localization.

In order to assess the impact of the p.Y142H mutation on GATA5 structural and functional properties, site‐directed mutagenesis was carried out on the corresponding coding region of the human GATA5 cDNA. Both the wild‐type and mutated cDNA were subcloned into a HA‐tagged expression vector and then sequenced before carrying overexpression assays in cultured mammalian cells. Transfection into HEK293 cells showed that both proteins were equally produced at the expected molecular weight (Fig. 2A), while transfection into both HEK293 and HeLa cells showed that nuclear localization of the protein was not altered (Fig. 2B).

### Reduced transcriptional activity of the GATA5 Y142H protein

**Figure 3:**
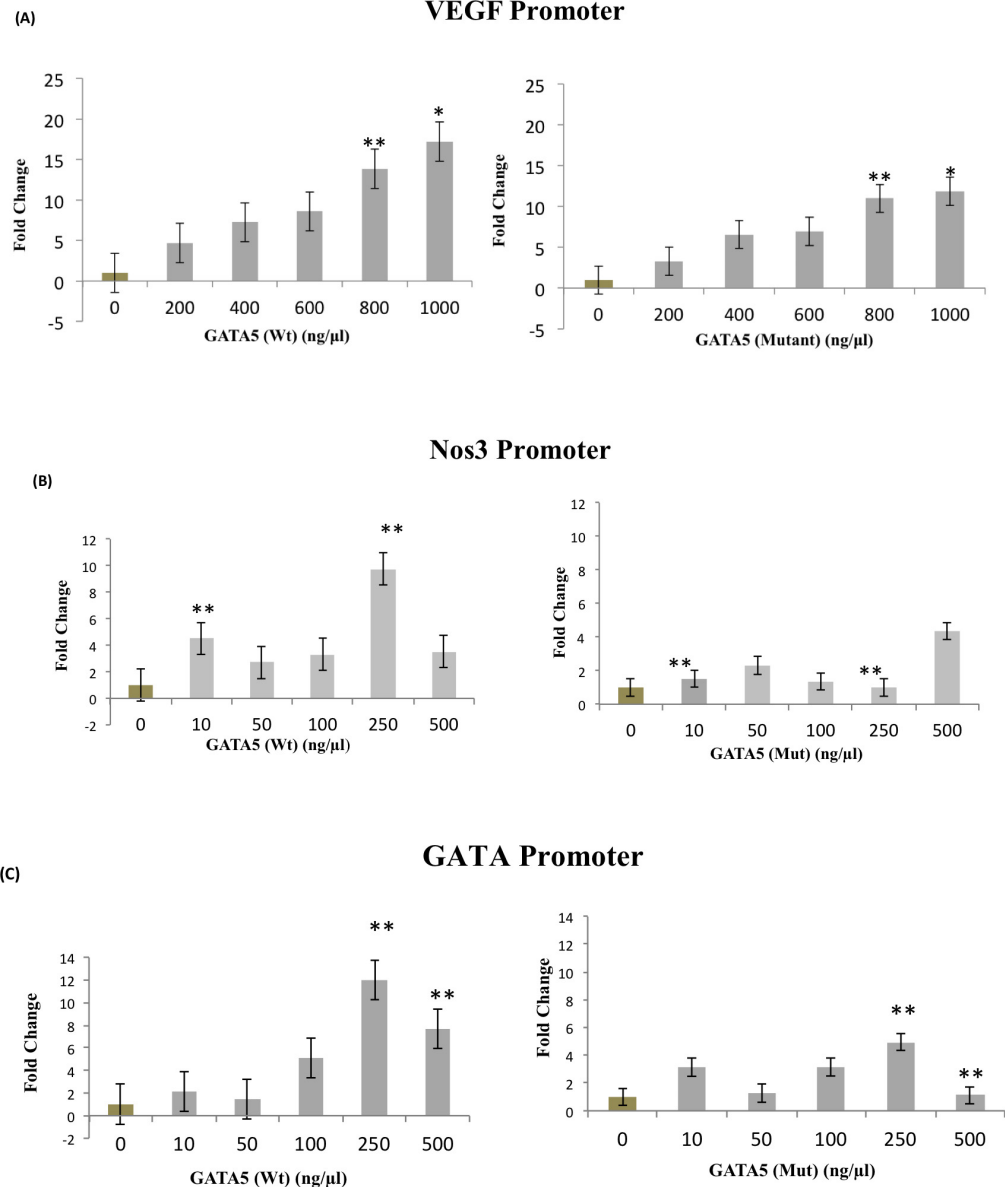
Transcriptional activity of the p.Y142H GATA5 protein Wt GATA5 or GATA5 mutant (Y142H) were transiently cotransfected with the human VEGF (A), or NOS3 (B), or the minimal GATA element (C) promoter coupled to luciferase in HeLa cells. Three hours posttransfection, media was changed and cells were harvested for luciferase assay after 36 h. Relative luciferase activities are represented as fold changes. The data are the means of 3 independent experiments done in duplicates ± SE. Significance (*P* < 0.05) was assessed using the one‐way ANOVA test. (*P < 0.01 mutant vs. wt, **P < 0.05 mutant vs. wt).

**Figure 4:**
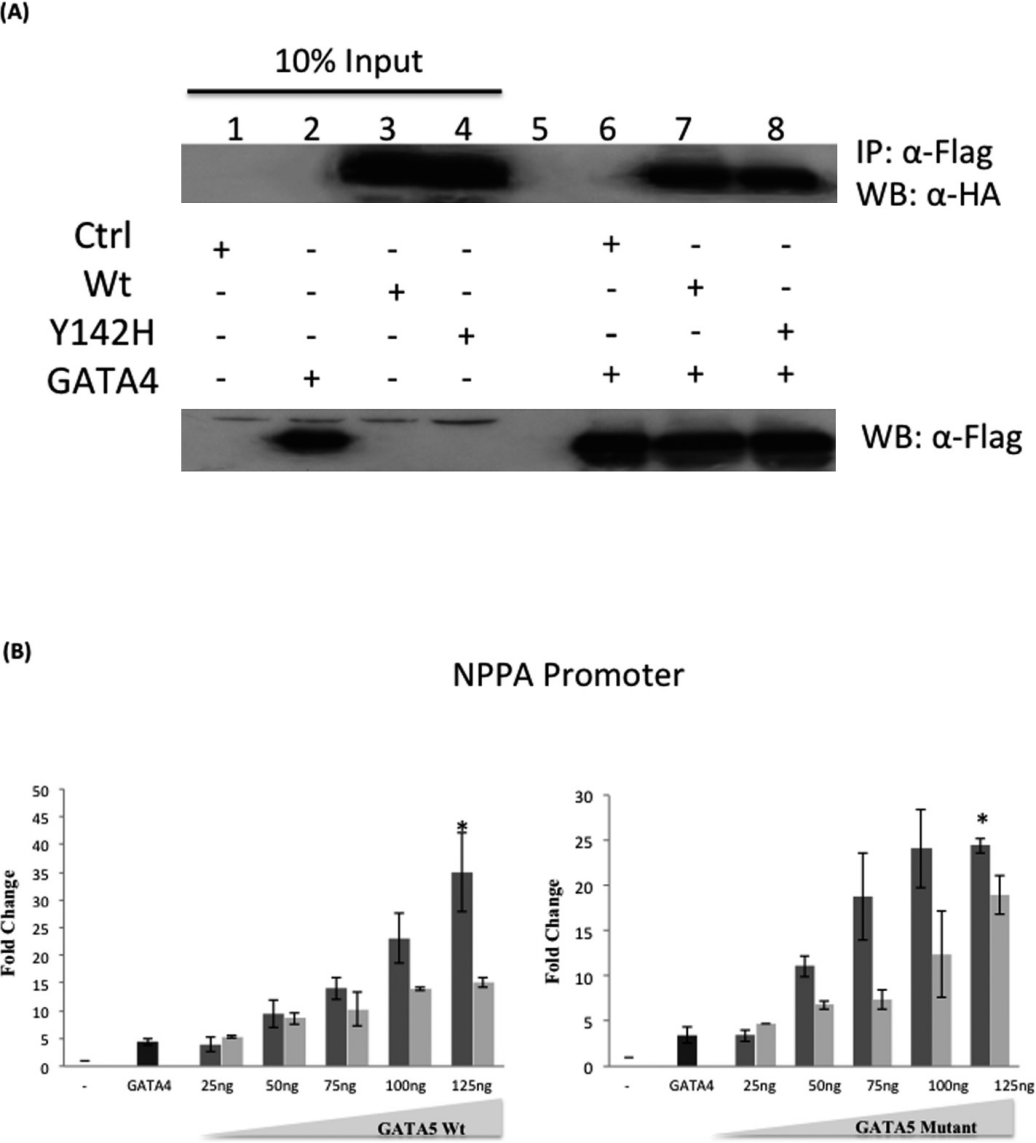
Effect of the p.Y142H mutation on the physical and transcriptional interaction between GATA4 and GATA5. 4A. Physical interaction between Flag‐tagged GATA4 and HA‐tagged GATA5 (Wt and Y142H) proteins is demonstrated (lanes 6,7, and 8). Ten times the quantity of proteins loaded for western blot was used for immunoprecipitation. Nuclear lysates of GATA5/complexes were immunoprecipitated with anti‐flag antibody and GATA5 proteins were visualized with western blot via anti‐HA antibody. Membrane stripping and subsequent western blot analysis was performed with anti‐Flag antibody in order to detect GATA4 proteins. 4B.Wt GATA5 or GATA5 mutant (Y142H) were transiently cotransfected with the rat NPPA‐luciferase promoter coupled to luciferase in HeLa cells. Three hours posttransfection, media was changed and cells were harvested for luciferase assay after 36 h. A fixed suboptimal dose of GATA4 was used along variable doses of GATA5 to assess possible functional interactions. Relative luciferase activities are represented as fold changes. The data are the means of three independent experiments done in duplicates ± SE. Significance (*P* < 0.05) was assessed using the one‐way ANOVA test. (*P < 0.01, **P < 0.05). Dark gray bars represent GATA5 wt or mutant alone, whereas light gray bars represent GATA5 wt or mutant with the fixed dose of GATA4.

In order to assess the effect of the p.Y142H mutation on the function of the *GATA5* gene, and given the fact that the evolutionary conserved Tyrosine residue is in the transactivation domain of the protein (Fig. 1), HeLa cells were cotransfected with increasing concentration of the *GATA5* wt or mutant plasmid, and the VEGF, Nos3, or NPPA‐luciferase‐fused promoters. Although the mutated protein was able to activate both promoters in a dose‐dependent manner, the maximum magnitude of this activation was consistently 30–35% less than the wild‐type protein (Figs. 3 and 4). This significant alteration of the transcriptional activity is independent of the promoter and cellular context, since it was also observed on an artificial GATA promoter harboring only one GATA element corresponding to the rat NPPB promoter (Fig. 3), and in Hek293 cells.

### Physical interaction with GATA5 partners not altered by the p.Y142H mutation

Given the fact that DORV is the major observed phenotype in the patient with homozygous expressivity of the variant p.Y142H allele, and based on the *Gata4/Gata5* double heterozygous mouse model which leads to the same phenotype, we assessed the possible physical and functional interaction between both proteins, and whether the mutated protein has any effect on this interaction. Coimmunoprecipitation assays showed that both GATA4 and GATA5 proteins are strong partners, but the p.Y142H mutation does not affect this interaction in a heterologous context which is the HEK293 cells (Fig. 4A). Additional assays with NFATC1 and Tbx5 did not yield any statistical variation between the wild‐type and mutated protein (data not shown). Cotransfection assays using the rat NPPA promoter did show, however, that GATA5 inhibits, in a dose‐dependent manner, the activation of GATA4 over the NPPA promoter reaching up to 30% inhibition and that the p.Y142H does not affect this inhibition (Fig. 4B). In sharp contrast with the previously described interaction between GATA4 and GATA6, we could not establish any synergistic interaction between GATA4 and GATA5 irrespective of the doses used for both expression vectors (data not shown). Instead, a constant dose of GATA4 inhibits GATA5 activation of the NPPA promoter in a dose‐dependent manner reaching up to 55% (Fig. 4B). This inhibtion was not however, affected by the p.Y142H variant. The same pattern of inhibition was also observed with a constant dose of GATA5 wt or mutant and increasing amounts of GATA4.

## Discussion

This study documents for the first time a direct link between a biallelic *GATA5* mutation in humans and a phenotype of double outflow right ventricle, VSD and PS. The genetic studies published on *GATA5* showed only heterozygous mutations as being linked to various forms of CHDs. This does not correlate however with the mouse genotype whereby only deletions of both alleles of Gata5 were needed to get a BAV phenotype which is not even completely penetrant. We have a total of eight variants in our screening of 185 patients with different forms of CHD, all inherited as heterozygous except for the p. T67P and p.Y142H variants.

### The GATA5 p.Y142H biallelic variant matches the mouse gentoype

The function of GATA5 during heart development was one of the most difficult to establish due to various confounding factors ranging from its time and space limited pattern of expression during development and ending with the existence of two functionally equipotent splice‐variants. The recent publication by Laforest et al. (2011) of the knock‐out model which lead to a partial penetrant BAV phenotype has opened the way to screen for genetic variants in patients with CHD that could explain the phenotype in mice. Since then a total of twenty missense variants were published (Fig. 1) as being associated with different forms of CHD ranging from BAV to AF, and including septal and valve defects (Gu et al. 2012; Jiang et al. 2012, 2013; Padang et al. 2012; Wei et al. 2012, 2013a,b; Yang et al. 2012; Wang et al. 2013; Bonachea et al. 2014; Shi et al. 2014; Zhang et al. 2014). None of the documented mutations, however, was homozygous. The heterozygosity of the *GATA5* mutational spectrum has been linked to the cardiac phenotypic abnormalities, and thus autosomal dominant pattern of inheritance has been postulated for the *GATA5* gene.

The common feature between all the published studies is the fact that the variants are heterozygous and only in rare cases the parents' genotypes were assessed. This makes it difficult to confirm association between genotypes and phenotypes although the minor allele frequencies were less than 3%. The only exception is the p.T252P variant which was documented by Shi et al. (2014) and which showed a clear inheritance pattern with a major defect in protein function since it affects the second zinc finger domain of GATA5. In most other cases, the inheritance pattern was not established. The p.G166S allele, for instance, was found to be associated with BAV in one Caucasian patient sharing the same phenotype with his mother, but in our case the same allele was found in a patient with TOF and another one with a single venticle (SV) phenotype (Table 2) (Padang et al. 2012). The two patients inherited this allele from one of their healthy parents. The p.S19W variant was also shown to be potentially associated with BAV, but in our study it was found in two unrelated patients one with ASD and another with TOF, and both inheriting it from a healthy parent (Table 2) (Padang et al. 2012). Despite being predicted to be damaging to the protein using both the PolyPhen2 (Adzhubei et al. 2010) and SIFT in silico bioinformatics tools, these variants cannot alone explain the observed phenotypes unless we take into consideration that other variants in genes within the same GATA5 pathway have also variants that undermine the function of GATA5. The three novel variants obtained in our screen ‐ p.R61S, p.G63A, and p.T289A ‐ are also heterozygous with no reported MAF in other populations. Our results, however, do show that they are inherited from one of the parents echocardiographically shown to be healthy. This does not definitely exclude any causality with the underlying phenotype since low penetrance is generally encountered in the genetics of CHD and at least for the p.T289A variant, it is predicted to be possibly damaging to the protein (Table 2). As for the p.Y142H variant, a previous report did correlate this genotype with BAV despite a lack of evidence of a family history. In our case, both parents who are first degree cousins are heterozygous for the genotype and echocardiograms did not show any cardiac defect, while the patient homozygous for the mutation has a DORV phenotype with ASD and PS. The genotype goes in parallel with the loss of function in mice whereby the cardiac BAV phenotype is linked to the total absence of the protein. This concordance with the mouse genetic model is reminiscent of other cardiac genes like *GATA4*,* GATA6*,* NKX2.5*, and *TBX5* whereby the genetic inheritance pattern is the same between humans and mice, although it is not a universal trend for all CHD cases (Elliott et al. 2003; Heinritz et al. 2005; Calderon Colmenero 2007; Tomita‐Mitchell et al. 2007; Nemer 2008; Maitra et al. 2010). In addition, the predicted in silico hampered functionality linked to this variant was corroborated by the fact that the amino acid is highly conserved in all species and more importantly is conserved between GATA4 and GATA5 (Morrisey et al. 1997a; Nemer et al. 1999). The position of the amino acid in the transactivation domain suggested that the mutation will affect the transcriptional activity of the protein. We did show that this activity was indeed reduced by 30–40% on all promoters tested and on an isolated GATA element. Similar experiments were previously done using a GATA4 variant changing the same amino acid into alanine, and showed a reduced activity of the protein by 60% confirming the evolutionary conserved role of this particular amino acid in GATA proteins (Morrisey et al. 1997b).

### GATA5 and DORV: is another variant needed to explain the phenotype?

Double outflow right ventricle is a complex phenotype that involves great vessel and valvular malposition in addition to septal anomalies accounting for approximately 1–3% of all CHDs. Parental consanguinity has been found to be significantly associated with familial forms of DORV, but only a couple of genes have been associated with the pathophysiology of the disease like *CFC1* (cripto, FRL‐1, cryptic family 1), and *ZFPM2* (zinc finger protein, multitype 2) (Goldmuntz et al. 2002; Tan et al. 2012). The phenotype is presented as a compilation of anatomical variants sharing similar pathophysiology where both of the great arteries connect to the right ventricle. Several classification systems have been proposed in the past in an attempt to unify various anatomical variants under the nomenclature of DORV including that of anatomic location of aortic valve in relation to VSD, and that of absence or loss of fibrous continuity between the aortic and mitral valve. Those definitions had their own drawbacks leading to the modern classification proposed by Van Praagh (1988) which describes primary malformations of the ventricular outflow portion of the heart (type I DORV), malformations of the ventricular OFT with additional malformations of the AV canal, AV valves, ventricles, venous and arterial pathways (type II), and lastly, defects in cardiac lateralization associated with DORV (type III). Our patient's phenotype belongs to the type II category, which includes among others aortic valve malformation, one of the phenotypes that could be linked to the mouse knock‐out model. Reduced transcriptional activities of the GATA5 p.Y142H protein could explain the phenotype taking into consideration the pattern of expression of GATA5 in the heart. However, the differences between the mice and human phenotypes might not be answered unless a knock‐in model is generated. The recently published mouse double heterozygous *Gata4/Gata5* model has exclusively a DORV phenotype prompting us to search for a potential link to our patient's phenotype/genotype correlation (Laforest and Nemer 2011). We screened for potential GATA4 variants in the patients by Sanger Sequencing of all coding exons but did not find any. We tested the possible interaction between GATA4 and GATA5 proteins and we found a strong physical interaction in vitro reminiscent of the GATA4/GATA6 interaction, however, the p.Y142H did not have any effect on this interaction. In parallel, and in sharp contrast with GATA6, GATA4, and GATA5 do not functionally interact to promote downstream transcriptional activity. They do rather compete in a dose‐dependent manner that could be due to a common cofactor, but once again the p.Y142H variant does not have any effect on the repression of GATA4 by GATA5.

In conclusion, we have documented the first *GATA5* mutation with an autosomal recessive pattern of inheritance that could be associated with the DORV phenotype.

## Conflict of Interest

All authors declare no competing financial interests.

## Supporting information


**Table S1.** Oligonucleotides used in the manuscript.
