# Supplementary material for: GATA5 mutation homozygosity linked to a double outlet right ventricle phenotype in a Lebanese patient
Source: Mol Genet Genomic Med. 2015 Dec 20;4(2):160–71. doi: 10.1002/mgg3.190 (PMC4799877; doi:10.1002/mgg3.190)
Supplement: Supplementary file 1 — Table S1. Oligonucleotides used in the manuscript. [file MGG3-4-160-s001.doc]

*GATA5 Mutation Homozygosity Linked to A Double Outlet Right Ventricle Phenotype in a Lebanese Patient*

Kassab K^1,#^, Hariri H^1,#^, Fahed AC^2^, Gharibeh L^3^, El-Zein M^1^, El- Rassi I^1^, Nemer M^3^, El-Rassy I^4^, Bitar F^1,5^, Georges Nemer^1,*^

Supplementary Table 1: Oligonucleotides Used in the Manuscript
